# Primary tumor-derived exosomes facilitate metastasis by regulating adhesion of circulating tumor cells via SMAD3 in liver cancer

**DOI:** 10.1038/s41388-018-0391-0

**Published:** 2018-07-10

**Authors:** Qihan Fu, Qi Zhang, Yu Lou, Jiaqi Yang, Gang Nie, Qi Chen, Yiwen Chen, Jingying Zhang, Jianxin Wang, Tao Wei, Hao Qin, Xiaowei Dang, Xueli Bai, Tingbo Liang

**Affiliations:** 1 Department of Hepatobiliary and Pancreatic Surgery, the Second Affiliated Hospital, Zhejiang University School of Medicine, Hangzhou, China; 2 Zhejiang Provincial Key Laboratory of Pancreatic Disease, Hangzhou, China; 3 Department of Medical Oncology, the Second Affiliated Hospital, Zhejiang University School of Medicine, Hangzhou, China; 4 Department of Hepatopancreatobiliary Surgery, the First Affiliated Hospital of Zhengzhou University, Zhengzhou, China

## Abstract

Hepatocellular carcinoma (HCC) is a fatal disease and patients with HCC frequently die from metastasis. The mechanisms of HCC metastasis are not completely understood. In the present study, in vitro and in vivo data showed that HCC cells promoted cancer cell proliferation and lung metastases formation in a paracrinal/endocrinal way. We found that HCC-derived exosomes mediated this phenomenon and observed enhanced cell adhesion in the presence of these malignant exosomes. We further identified that reactive oxygen species (ROS) regulated the adhesive molecules. Intriguingly, attached HCC cells released exosomes containing both SMAD Family Member 3 (SMAD3) protein and mRNA, which were delivered to detached HCC cells and facilitated their adhesion. These exosomes induced enhanced SMAD3 signaling in the recipient HCC cells and increased their adhesive ability. In addition, we showed that SMAD3-abundant exosomes existed in the peripheral blood of patients with HCC, and their levels correlated with disease stage and the SMAD3 expression of primary tumors. Our study suggested a possible mechanism by which primary HCC supported metastases formation and revealed the role of SMAD3 in the exosomes-mediated crosstalk between primary and circulating HCC cells.

## Introduction

Hepatocellular carcinoma (HCC) is one of commonest types of cancer worldwide, especially in developing countries, including China. Most patients with HCC die from tumor metastasis, the mechanisms of which remains unclear. The latest metastasis model demonstrates that metastases arise from circulating tumor cells (CTCs), which originate from primary tumors. However, the relationship between the primary tumor and metastases is not clear. Although the argument concerning tumor homeostasis has lasted for more than a century, clinical investigations have demonstrated that surgery-driven enhancement of metastasis development may be case-dependent [1]. Provided that only single CTCs or CTC clusters containing a few cancer cells extravasate out of the vessel in a particular site, in addition to the attack of local immune cells, the survival of these cancer cells is difficult [2]. It is thus hypothesized that the primary tumor might provide additional support for metastases formation. Recent studies have provided evidence for this hypothesis. For instance, primary tumor-derived exosomes (PTDEs) can create a pre-metastatic niche in pre-determined metastatic organs by inducing immunosuppression, fibrosis, or inflammation [3–5]. However, few studies have focused on the effects of PTDEs on CTCs.

Attachment of CTCs to the lining of the microvasculature is an indispensible step for cancer cell extravasation and subsequent metastasis formation [6]. Interference with CTC adhesion impairs successful CTC seeding and colonization [7]. Reactive oxygen species (ROS) are crucial regulators of cell adhesion [8], and an increased ROS level was reported in CTCs [9]. A high ROS level is associated with enhanced invasiveness and metastasis in HCC [10, 11]. However, in circulating HCC cells, the regulation of ROS and CTC adhesion are largely unknown.

Exosomes are a group of vesicles secreted by most cell types in vivo and in vitro, with a diameter of ~ 50 nm [12]. They harbor numerous biological macromolecules, including proteins and RNA, which can be transferred between cells [13]. In circulation, CTCs and PTDEs have an increased opportunity to contact with each other. Thus, PTDEs-mediated communication between the primary tumor and CTCs is possible. The mechanisms of such communication are currently poorly understood. In the present study, using in vivo and in vitro models, we showed that PTDEs promote lung metastases formation by regulating CTC proliferation and adhesion. Mechanistically, we revealed a PTDE-mediated SMAD Family Member 3 (SMAD3)-ROS signaling pathway to induce cell adhesion.

## Results

### Primary tumors promote lung metastasis

**Fig. 1:**
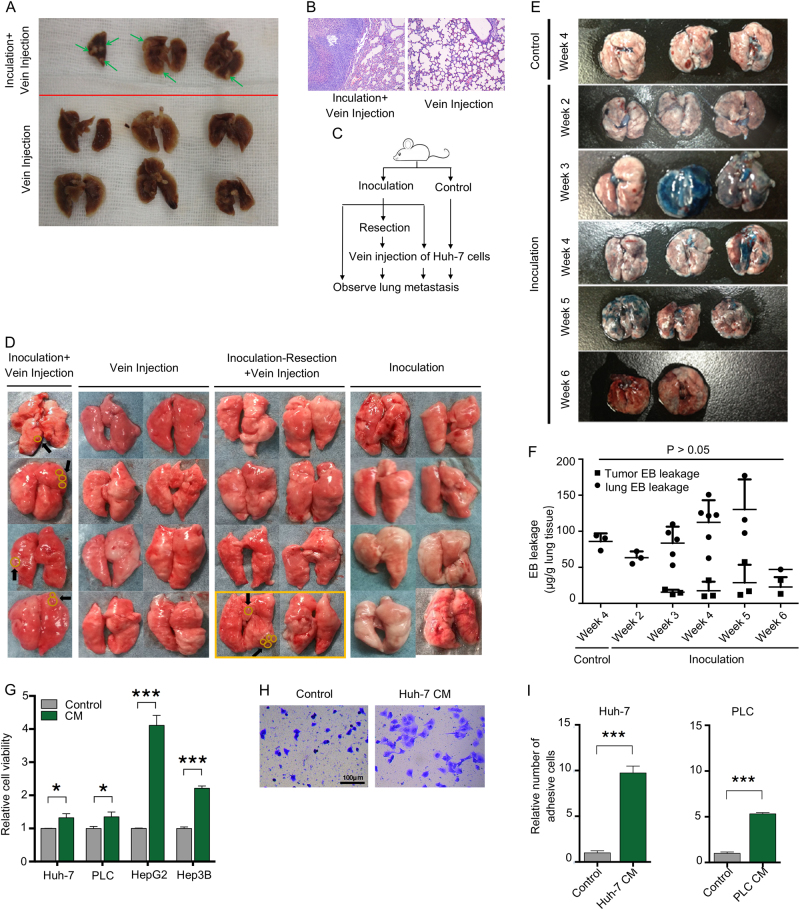
Primary tumors promote lung metastasis. **a**, **b** Huh-7 cells were intravenously injected into mice with or without subcutaneous inoculation of Huh-7 cells. Lung metastasis was examined after 4 weeks. Green arrows indicate metastatic nodules. Pathology analysis confirmed that the nodules in lungs were metastatic lesions. **c**, **d** Mice were treated as indicated, and gross lung metastasis was examined. Yellow circles indicate metastatic lesions. **e**, **f** Mice, with or without Huh-7 cells inoculation, were injected with Evans Blue (EB) via the caudal vein at the indicated time. Lungs were photographed, and EB leakage in the lungs and tumors were measured. **g** HCC cells were cultured in fresh medium or HCC conditioned medium for 2 h. Cell viability was assessed using CCK-8 assays. **h**, **i** HCC cells were cultured with fresh medium or Huh-7-conditioned medium for 2 h. The adhesive cells were photographed and counted. Representative images are shown. **f**, **g**, **i** Data are expressed as means ± SD of at least three independent experiments. Statistical analysis was made using one-way ANOVA **e** or an unpaired *t* test **f**, **h**. *P < 0.05, ***P < 0.001

To investigate whether primary tumors provide other support for metastasis formation in addition to metastatic seeds (i.e., CTCs), we injected Huh-7 cells via the caudal vein into mice with or without in-advance subcutaneous inoculation of the same HCC cell line. After 4 weeks, we observed lung metastasis in all mice with subcutaneous xenografts, but none in those without tumor inoculation (*P* < 0.05; Fig. 1a, b). To confirm this phenomenon, we designed a more complicated experiment (Fig. 1c), which showed that all mice with subcutaneous tumors had lung metastases, whereas those with merely subcutaneous inoculation or caudal vein injection of HCC cells did not (Fig. 1d and Table 1). Intriguingly, we completely removed the subcutaneous tumors from eight mice 2 days before caudal vein injection of HCC cells, and no lung lesions were found in six of them, without tumor recurrence. However, the other two mice with tumor inoculation and resection suffered tumor recurrence because of incomplete removal. Notably, one of the two mice suffered from lung metastasis. No liver or brain metastases were found in these experiments. These results indicated that the presence of a subcutaneous tumor facilitated lung metastasis.

**Table 1:** Lung metastasis in the primary tumor-CTC mouse model

	Incidence	No. of nodules^a^
Inoculation	0/8	0
Vein injection	0/8	0
Inoculation + vein injection	4/4^b^	1.8 (1–3)
Inoculation-resection + vein injection	0/6	0
Post-resection recurrence	½	2.5 (0–5)

^a^Data are shown as the average (range)

^b^*P* < 0.01 compared with other groups, except the post-resection recurrence group

To understand the underlying mechanisms by which primary tumors promote lung metastasis formation, we first considered the possible enhancement of pulmonary vascular permeability caused by the subcutaneous xenograft. Although a trend of increased vascular leakage in the lungs of mice was observed with increased time, no significant alteration was observed (Fig. 1e, f). We then assumed that primary tumors might directly affect the biology of CTCs by enhancing their survival, proliferation, or adhesion, which are all critical for successful metastasis formation. In an in vitro model, we found that tumor cell conditioned medium enhanced tumor viability and adhesion to a greater extent than fresh medium (Fig. 1g–i), suggesting that tumor cell-derived bioactive materials in the medium may support the metastasis-related characteristics of HCC cells.

### PTDEs mediates cancer cell proliferation and adhesion

**Fig. 2:**
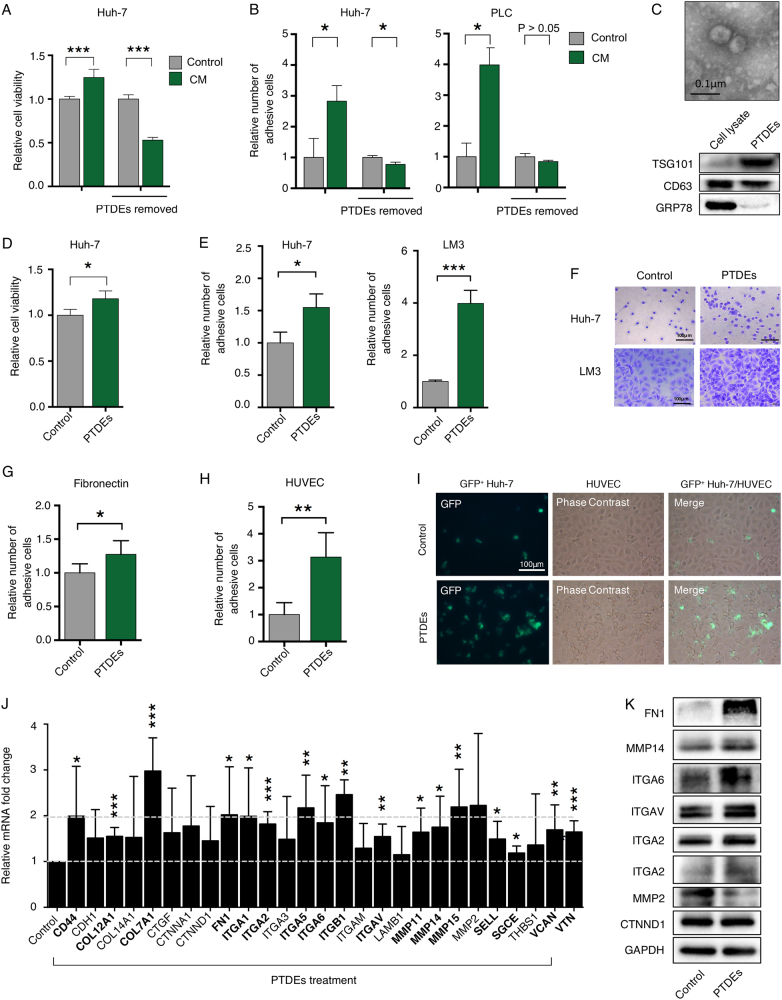
PTDEs mediate cell proliferation and adhesion. **a** Huh-7 cells were cultured in indicated medium with or without PTDEs removal for 2 h. Cell viability was determined using CCK-8 assays. **b** Huh-7 and PLC cells were cultured in the indicated media for 2 h. Number of adhesive cells were counted and compared. **c** Visualization of exosomes by transmission electron microscopy. Enriched TST101 and CD63 (exosomes markers) were detected in PTDEs, GRP78 (an endoplasmic reticulum marker) was almost negative. **d** Huh-7 cells were cultured and treated with or without PTDEs. **e**, **f** Huh-7 or LM3 cells were cultured and treated with or without PTDEs. **e** Statistical analysis of adhesive Huh-7 and LM3 cells exposed to PTDEs. **f** Representative images for Huh-7 and LM3 cells are shown. **g** Huh-7 cells were cultured on fibronectin-coated plates and treated with PTDEs for 2 h. Statistical analysis of adhesive cells are shown. **h**, **i** GFP^+^ Huh-7 cells were cultured on HUVECs-coated plates and treated with PTDEs for 2 h. Representative images and statistical analysis of GFP^+^ adhesive cells are shown. **j** The relative mRNA levels of indicated genes were measured by qRT-PCR in detached Huh-7 cells exposed to PTDEs for 6 h compared with the cells that did not receive PTDEs. **k** The expression of indicated proteins were measured by western blotting in detached Huh-7 Cells exposed to PTDEs for 6 h compared with the cells that did not receive PTDEs. **a**, **b**, **d**, **e**, **g**, **h**, **j** Data are expressed as means ± SD of at least three independent experiments. Statistical analysis was performed using unpaired *t* test. *P < 0.05, **P < 0.01, ***P < 0.001

Given the importance of tumor-derived exosomes in cancer metastasis [14], we hypothesized that PTDEs contributed to tumor metastasis by directly regulating the survival and adhesion of CTCs. After removing exosomes from the conditioned medium, we observed reduced effects in terms of cell viability and adhesion (Fig. 2a, b). We then isolated exosomes from Huh-7-conditioned medium (Fig. 2c, d), and used them to treat Huh-7 cells. As expected, PTDEs enhanced cell viability of Huh-7 cells (Fig. 2d). PTDEs also significantly enhanced the cell adhesion in both Huh-7 and LM3 cells (Fig. 2e, f). This phenomenon was further verified in fibronectin- and human umbilical vascular endothelial cells (HUVECs)-coated plates (Fig. 2g–i). In support of these results, the expression of many cell adhesion-related molecules was upregulated in the presence of PTDEs (Fig. 2j, k). Collectively, we demonstrated that PTDEs were capable of promoting cell viability and more importantly, cell adhesion.

### Delivery of bioactive materials from primary tumors to CTCs by PTDEs

**Fig. 3:**
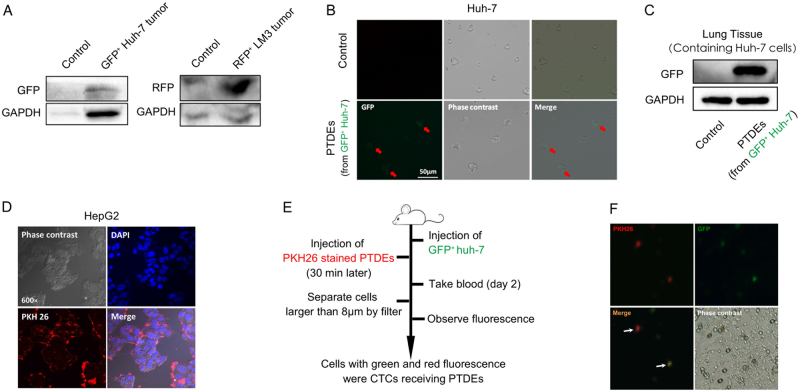
Intercellular delivery of materials by PTDEs. **a** Mice were subcutaneously inoculated with GFP^+^ Huh-7 cells or RFP^+^ LM3 cells. Exosomes were isolated from the mice sera 2 weeks later. GFP and RFP were detected in the exosomes, respectively. **b** Naive Huh-7 cells were incubated with GFP^+^ Huh-7 cell-derived exosomes for 24 h. GFP was detected in the recipient cells by fluorescence. **c** Mice were intravenously injected with naive Huh-7 cells and GFP^+^ Huh-7-derived PTDEs successively. Mice were killed 2 h later, and GFP was detected in the lungs that contained intercepted Huh-7 cells. **d** HepG2 cells were incubated with PKH26-labeled exosomes derived from HepG2 cells for 2 h. Fluorescence was performed to identify nuclei (blue) and PKH26-labeled cell membrane (red). **e**, **f** Mice were treated as indicated. PKH26 (red)-labeled GFP^+^ Huh-7 cells were isolated from the mice circulation

To confirm that PTDEs can be directed to CTCs, we first tested the existence of PTDEs in mouse circulation. We subcutaneously inoculated green fluorescent protein (GFP)^+^ Huh-7 cells or red fluorescent protein (RFP)^+^ LM3 cells into mice, and isolated exosomes from mouse blood. Both GFP and RFP could be detected in serum exosomes (Fig. 3a), indicating the existence of PTDEs in our mouse model. We then incubated naive Huh-7 cells with GFP^+^ Huh-7-derived PTDEs, and successfully observed GFP signals in the recipient Huh-7 cells (Fig. 3b). To demonstrate that delivery of bioactive materials from tumor-derived exosomes to CTCs in vivo was possible, we injected naive Huh-7 cells and GFP^+^ Huh-7-derived PTDEs into mice successively, and detected GFP from the lungs that contained intercepted Huh-7 cells (Fig. 3c). Similarly, when we incubated receiver HepG2 cells with PKH26-labeled PTDEs that extracted from donor HepG2 cells, red fluorescence in the receiver cells was observed (Fig. 3d), suggested that exosomes could fuse with tumor cells and probably deliver the materials in PTDEs into tumor cells. In addition, we injected GFP^+^ Huh-7 cells into blood stream to mimic CTCs, followed by injection of PKH26 stained PTDEs, to test the possible delivery of bioactive materials in vivo (Fig. 3e). As expected, PKH26-labeled GFP^+^ cells were isolated from mouse blood (Fig. 3f), suggesting direct material delivery from PTDEs to CTCs. Taken together, these results implicated that primary tumors could delivery bioactive materials to CTCs through PTDEs.

### PTDEs promote cancer cell adhesion by enhancing ROS

**Fig. 4:**
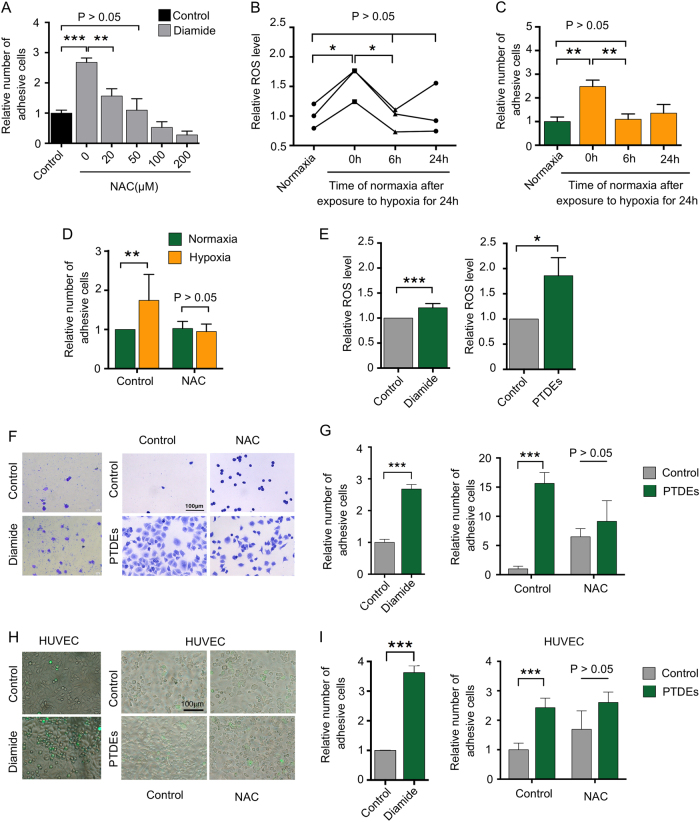
PTDEs promote cancer cell adhesion by enhancing ROS. **a** Huh-7 cells were cultured on a 96-well plate and treated with diamide (100 μM) and different concentrations of NAC for 24 h. Adhesive cells were counted. **b**, **c** The ROS level in Huh-7 cells and relative number of adhesive Huh-7 cells were measured in normoxia or hypoxia. **d** Huh-7 cells were exposed to hypoxia or normoxia in presence or absence of NAC (100 μM). Adhesive cells were counted. **e** Huh-7 cells were incubated with PTDEs and the ROS level in Huh-7 cells was measured. **f**, **g** Huh-7 cells were cultured on a 96-well plate and incubated with PTDEs in the presence or absence of NAC (100 μM) for 6 h. **f** Representative images are shown. **g** Relative numbers of adhesive cells were calculated. **h**, **i** GFP^+^ Huh-7 cells were cultured in HUVECs-coated plates and incubated with PTDEs in the presence or absence of NAC (100 μM) for 6 h. **h** Representative images are shown. **i** Relative numbers of adhesive cells were calculated. **a**–**e**, **g**, **i** Data are expressed as means ± SD of at least three independent experiments. Statistical analysis was performed using the unpaired *t* test. *P < 0.05, **P < 0.01, ***P < 0.001

We then explored how PTDEs regulated cell adhesion in our setting. ROS are a key modulators of cell adhesion [8]; therefore, we determined the ROS level in Huh-7 cells with or without PTDEs incubation. The ROS inducer diamide significantly increased cell adhesion, which could be inhibited by N-acetyl cysteine (NAC), a ROS scavenger, in a dose-dependent manner (Fig. 4a). Hypoxia significantly upregulated the ROS level and the cell adhesion capacity of Huh-7 cells (Fig. 4b, c), and NAC eliminated this hypoxia-induced cell adhesion (Fig. 4d). Notably, PTDEs from Huh-7 cells enhanced the ROS level and cell adhesion of the recipient cells in different adhesive conditions (Fig. 4e–i). However, in the presence of NAC, the pro-adhesive effect of PTDEs was abolished (Fig. 4f–i). These findings suggested that ROS could be a key factor that mediates PTDE-induced cell adhesion.

### PTDEs induce ROS in recipient HCC cells by enhancing SMAD3 signaling

**Fig. 5:**
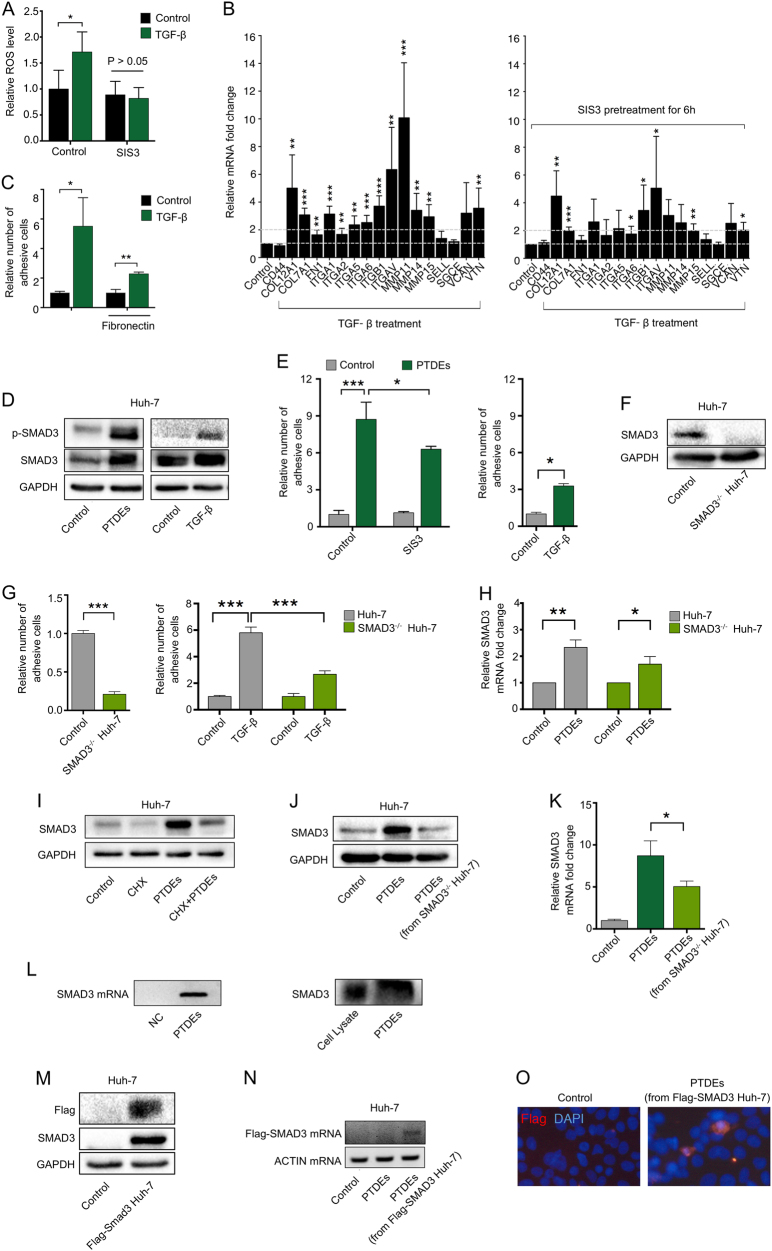
PTDEs upregulate ROS in recipient cancer cells by enhancing SMAD3 signaling. **a**, **b** Huh-7 cells were cultured with TGF-β (5 ng/ml) with or without SIS3 (3 µM) pretreatment for 6 h. **a** The ROS level was measured. **b** The relative mRNA levels of indicated genes were measured using qRT-PCR. **c** Huh-7 cells were cultured on normal plates and fibronectin-coated plates and treated with TGF-β (5 ng/ml) for 6 h. Adhesive cells were counted. **d** Naive detached Huh-7 cells were incubated with PTDEs for 6 h, and the expression of SMAD3 and p-SMAD3 was detected. **e** Huh-7 cells were incubated with PTDEs in the presence or absence of SIS3 (3 µM) pretreatment. Adhesive cells were counted. **f** SMAD3 protein was not detectable in *SMAD3*^−/−^ Huh-7 cells. **g** Wild-type and *SMAD3*^−/−^ Huh-7 cells were cultured on a 96-well plate for 2 h, and adhesive cells were counted in presence or absence of TGF-β. **h** Wild-type and *SMAD3*^−/−^ Huh-7 cells were incubated with PTDEs for 6 h, and the mRNA level of *SMAD3* was measured by qRT-PCR. **i** Huh-7 cells were treated with cycloheximide (CHX; 50 µg/ml) and/or PTDEs for 6 h. *SMAD3* expression was detected. **j**, **k** Huh-7 cells were cultured on a 96-well plate and treated with PTDEs from naive or *SMAD3*^−/−^ Huh-7 cells for 6 h. **j** *SMAD3* expression was detected. **k** The mRNA level of *SMAD3* were measured by qRT-PCR. **l** The existence of *SMAD3* mRNA and protein in PTDEs was evaluated by reverse transcription PCR and western blotting, respectively. **m** The expression of FLAG and SMAD3 was detected in FLAG-SMAD3 stably transfected Huh-7 cells by western blotting. **n**, **o** Huh-7 cells were incubated with PTDEs from naive or FLAG-SMAD3 Huh-7 cells for 6 h. **n** The existence of *FLAG-SMAD3* mRNA product was evaluated by reverse transcription PCR. **o** The FLAG-SMAD3 fusion protein was detected by immunofluorescence. **a**–**c**, **e**, **g**, **h**, **k** Data are expressed as means ± SD of at least three independent experiments. Statistical analysis was performed using the unpaired *t* test. *P < 0.05, **P < 0.01, ***P < 0.001

Tumor growth factor (TGF)-β/SMAD signaling induces ROS via NADPH oxidase [15]. In our model, we confirmed that TGF-β could increase the ROS level and upregulate the expression of various adhesion-related molecules (Fig. 5a, b). Notably, these effects could be blocked by SIS3, a SMAD3 inhibitor. In addition, TGF-β also enhanced cancer cell adhesion (Fig. 5c). These results suggested a close association between TGF-β signaling and ROS-mediated cell adhesion. SMAD3 is a critical component in TGF-β signaling; therefore, we assessed SMAD3 levels, and found overexpression of SMAD3 and phosphorylated SMAD3 (p-SMAD3) in the recipient cells treated with PTDEs (Fig. 5d). In addition, treatment with SIS3 in the recipient Huh-7 cells impaired the adhesion-enhancing effect of PTDEs (Fig. 5e), indicating an important role of SMAD3 in PTDEs-mediated cell adhesion. As a further validation, *SMAD3*^−/−^ Huh-7 cells showed a reduced cell adhesion ability, even in the presence of TGF-β (Fig. 5f, g), suggesting that SMAD3 plays a key role in HCC cell adhesion. In addition, PTDEs treatment led to an increase in the mRNA levels of *SMAD3* in the recipient Huh-7 cells, even in SMAD3^−/−^ cells without endogenic SMAD3 mRNA (Fig. 5h). Notably, by blocking mRNA translation in the recipient cells with cycloheximide, we observed a reduced, but not eliminated effect, of PTDEs to increase the SMAD3 protein level in the targeted cells (Fig. 5i), suggesting direct delivery of both SMAD3 mRNA and protein by PTDEs. In agreement this observation, PTDEs from *SMAD3*^−/−^ Huh-7 cells had no influence on the SMAD3 level in the recipient cells and consequently showed reduced effects on cell adhesion (Fig. 5j, k). In addition, both SMAD3 mRNA and protein could be detected in PTDEs (Fig. 5l). To further validate the independent delivery of SMAD3 mRNA and protein via PTDEs, we forced Huh-7 cells to express FLAG-tagged SMAD3 (Fig. 5m), and incubated naive Huh-7 cells with exosomes from these cells. As expected, both the mRNA and protein of the exogenous FLAG-tagged SMAD3 were detected in the recipient cells (Fig. 5n, o). Taken together, these results showed that PTDEs promoted cell adhesion by enhancing SMAD3 signaling in the recipient cells by supplying both SMAD3 mRNA and protein.

### SMAD3-containing exosomes are increased in HCC patients and correlate with the clinicopathology of patients

**Fig. 6:**
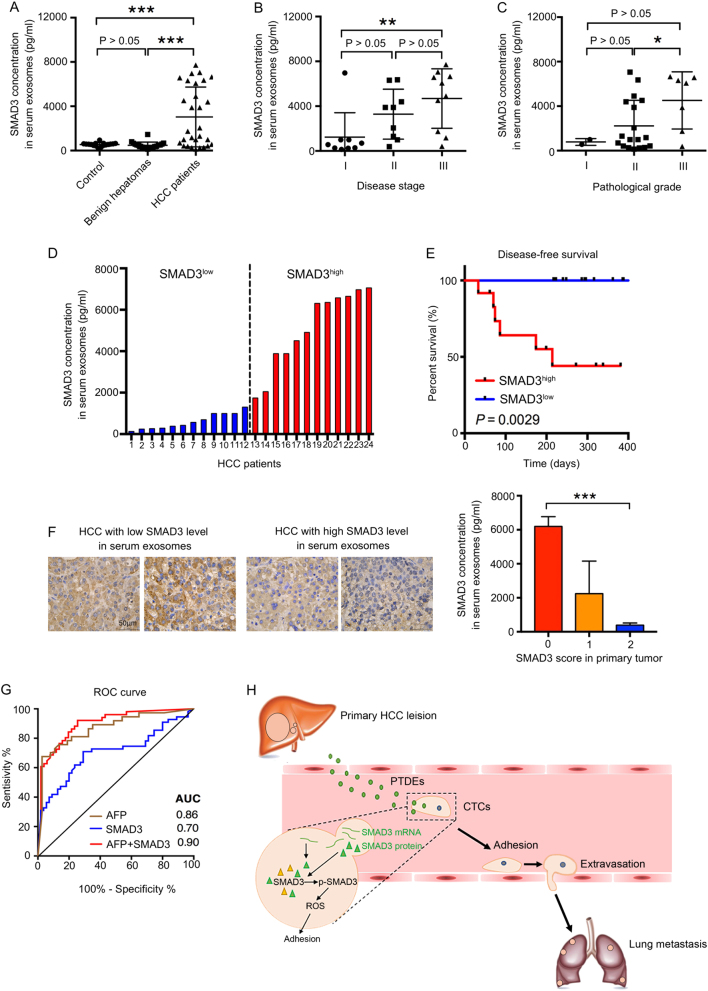
SMAD3-containing exosomes increase in HCC patients’ sera and correlate with the clinicopathology of HCC patients. **a** The level of SMAD3 protein in the serum exosomes derived from healthy donors and patients was determined by ELISA. **b**, **c** The SMAD3 level in HCC patients’ serum exosomes was correlated with disease stage and pathological grade. **d** Grouping of 24 HCC patients cutoff by the median value (1523 pg/ml). **e** Disease-free survival of HCC patients with high (*n* = 12) or low (*n* = 12) SMAD3 levels in serum exosomes was estimated using the Kaplan–Meier method. **f** The expression of SMAD3 was determined in HCC tissue. Representative images are shown from four patients with low and high SMAD3 levels in serum exosomes. Statistic analysis of SMAD3 concentration in serum exosomes vs. in HCC tissues was shown (*n* = 11). **g** The receiver operating characteristic (ROC) curve of AFP and/or exosomes-containing SMAD3 for HCC diagnosis. HCC group (*n* = 29), Control group (*n* = 37). **h** A scheme showing that PTDEs promote lung metastasis by enhancing adhesion of CTCs. Primary HCC lesions release PTDEs containing both mRNA and protein of SMAD3. The SMAD3-containing PTDEs deliver the bioactive materials including the *SMAD3* mRNA and protein to CTCs in the circulation. Enhanced SMAD3 signaling induces a higher level of ROS in CTCs. The adhesive capacity of CTCs is upregulated by increased ROS, and thus more CTCs can extravasate to form metastases in the lung

To understand the clinical value of our findings, we evaluated SMAD3-containing exosomes in human peripheral blood. Compared with healthy donors and patients with benign hepatomas, patients with HCC had significantly elevated levels of SMAD3-containing exosomes (Fig. 6a). Further analysis suggested that the abundance of SMAD3-containing exosomes correlated positively with both disease stage and pathological grade in patients with HCC (Fig. 6b, c). More importantly, the abundance of SMAD3-containing exosomes correlated negatively with disease-free survival of patients with HCC post surgery (Fig. 6d, e). Intriguingly, we found that a high level of SMAD3-containing exosomes correlated negatively with the expression of SMAD3 in the primary tumors (Fig. 6f), suggesting that primary tumors discarded SMAD3 via PTDEs. In addition, combined detection of SMAD3-containing exosomes and α-fetoprotein improved the diagnosis of HCC in humans (Fig. 6g). Therefore, a blood test for SMAD3-containing exosomes may have both diagnostic and prognostic values.

## Discussion

Tumor homeostasis is a crucial issue because it is not only a fundamental problem of tumor biology, but also is closely related to the clinical treatment of patients. In the clinic, patients with distant metastasis usually have no opportunity for surgery, because most surgeons believe that there is very limited benefit to gain from operating on these patients. Only in a few clinical scenarios, such as colorectal cancer with liver metastasis and breast cancer with lung/liver/brain/sternum metastasis, can primary tumor resection be considered [16, 17]. This opinion is supported by the hypothesis that numerous cancer cells have been already seeded in the whole body once imaging of detectable metastasis is found. The majority of metastatic cancer cells do not proliferate rapidly because they are dormant until recovery induced by suitable stimulators. In agreement with this hypothesis, clinical resection of primary tumors was reported to induce metastatic growth in some cases [18, 19]. Using animal models, investigators reported that primary tumors could inhibit angiogenesis and promote apoptosis of cancer cells in metastases [18, 20]. This hypothesis is reasonable, because most patients with cancer die from metastasis, and dissemination of cancer cells happens in the very early stage of tumor development [21]. Thus, resection of a primary tumor seems unable to remove seeded cancer cells, and even worse, may stimulate the outgrowth of dormant cancer cells. However, with advances in cancer treatment and the extension of patients’ survival, this traditional opinion should be reconsidered. Patients with resected primary tumors might benefit from a reduced risk of late-stage metastasis. Indeed, recently, increasing reports on primary resection in patients with metastasis of different types of cancer have noted good survival [22–24]. Promising results of primary tumor resection in patients with HCC with resectable metastatic lesions are also emerging [25]. Therefore, an understanding of primary tumor-supported metastasis formation is important to balance the benefit and harm in decision-making regarding primary tumor resection.

We proved that primary tumors could aid CTCs by enhancing their survival and adhesion. We found that single HCC cells in flow culture conditions that mimicked the bloodstream in vivo showed minimal proliferation (data not shown). In addition, the time window during which CTCs can be detected in circulation is only several minutes to hours [2]. We thus focused our study on cell adhesion using in vitro models. PTDEs were observed to enhance cell adhesion by inducing ROS via SMAD3 signaling. We initially tested PTDEs from both normoxic and hypoxic HCC cells, considering the different oxygen conditions in primary tumors. Although both normoxic and hypoxic Huh-7-derived exosomes showed strong induction of ROS and cell adhesion, normoxic cells-derived exosomes showed better results. This may be because of reduced SMAD3 expression in primary tumor cells exposed to hypoxia, as we have demonstrated previously [26]. These results suggested that tumor cells located in places with a relatively abundant vasculature might contribute more to primary tumor-supported CTCs adhesion.

Studies have demonstrated the roles of exosomes in promoting HCC progression [27, 28]. In the present study, we confirmed the pro-tumoral effects of exosomes. In particular, we provided a novel mechanism of SMAD3 signaling crosstalk between HCC cells, or more particularly, between primary tumors and CTCs (Fig. 6h). As far as we know, this is the first study to show that SMAD3 protein and mRNA can be transferred between cancer cells spontaneously, highlighting this signaling in HCC metastasis. In addition, other exosome-mediated mechanisms of SMAD3 activation were observed. For example, we found that αV integrin (ITGAV) also played a role in our model, because exosomes secreted by *ITGAV*-knockout Huh-7 cells showed a weaker potentials for SMAD3 activation and cell adhesion capacity of the recipient cells (data not shown). Thus, the SMAD3 signaling between primary tumors and CTCs could be more complicated than that revealed by the present study.

TGF-β/SMAD signaling has a dual role in tumor development [29]. The tumor suppressive or oncogenic effect of TGF-β in HCC is tumor stage-dependent. Coulouarn et al. [30] showed that a late-stage TGF-β signature with reduced SMAD3 signaling was associated with enhanced tumor invasive capacity and an increased tumor recurrent rate. The negative correlation of primary tumor and serum exosomes in terms of SMAD3 levels in our study implies that highly malignant primary tumors tend to release SMAD3 through exosomes. This action may benefit both the primary tumor cells and CTCs by increasing invasiveness and enhancing adhesion, respectively. Although different phosphoisoforms of SMAD3 can have opposite prognostic roles in HCC [31], detection of exosomes involves only total SMAD3 and does not require determination of specific phosphoisoforms. More importantly, the expression of SMAD3 and its different phosphoisoforms may have location heterogeneity in primary tumors; however, serum exosomes, as a liquid biopsy substrate, reflect the comprehensive expression of SMAD3 in all cancer cells of primary tumors. Furthermore, we provided limited, but promising, evidence for a better diagnostic and prognostic tool using SMAD3-containing exosomes. Unfortunately, because of the short follow-up duration, we were unable to test the predictive value of SMAD3-containing exosomes for overall survival in patients with HCC. Therefore, the clinical value of serum SMAD3-containing exosomes requires further study.

In conclusion, we demonstrated that exosomes-enhanced SMAD3/ROS signaling induced cell adhesion in HCC cells. We proposed a model for crosstalk between primary tumors and CTCs via PTDEs, and also suggested the possible clinical value of serum SMAD3-containing exosomes in patients with HCC.

## Materials and methods

### Cell culture and reagents

Huh-7 cells (a human HCC cell line) were purchased from the Japanese Collection of Research Bioresources (JCRB) Cell Bank (Ibaraki Osaka, Japan). HepG2 cells (a human HCC cell line) and HUVECs were purchased from American Type Culture Collection (ATCC; Manassas, VA, USA). LM3 cells (a mouse breast cancer cell line) were purchased from the Cell Bank of Shanghai Institutes for Biological Sciences (Shanghai, China). GFP^+^ Huh-7 cells were generated by stably transfecting Huh-7 with GFP-encoding plasmids (GenePharma, Shanghai, China). *SMAD3*^−/−^ Huh-7 cells were generated by Crispr/Cas9 genome editing. FLAG-SMAD3 Huh-7 was generated by stably transfected with lentiviruses containing a FLAG-SMAD3 encoding sequence (Obio Technology, Shanghai, China). Huh-7, HepG2, and LM3 cells were cultured in Dulbecco’s Modified Eagle Medium (DMEM; Gibco, Thermo Fisher Scientific, Waltham, MA, USA). HUVECs were cultured in Roswell Park Memorial Institute (RPMI)-1640 medium (Gibco). Both media were supplemented with 10% fetal bovine serum (FBS; Gibco) and 1% penicillin/streptomycin (Gibco). For hypoxia treatment, cells were cultured in a hypoxic incubator with 1% O_2_ for 24 h.

Diamide, NAC, and PKH26 Red Fluorescent Cell Linker Mini Kit were purchased from Sigma-Aldrich (St. Louis, MO, USA). The SMAD3 inhibitor SIS3 was purchased from Santa Cruz Biotechnology(CAS 1009104-85-1; CA, USA)

### Exosome extraction

Exosomes were isolated by a precipitation method using ExoQuick (System Biosciences, Palo Alto, CA, USA) according to the manufacturer’s instructions. In brief, HCC cells were seeded in 10-cm dishes. When the cells reached 90% confluence, culture medium was replaced with FBS-free DMEM. After 24 h of culture, supernatants were collected and referred as tumor conditioned medium. The conditioned medium was subjected to centrifugation at 10,000 × *g* for 1 h to remove large cellular debris. Afterwards, the supernatants were further subjected to ultra-filtration centrifugation (Amicon Ultra Centrifugal Filters, 3000 NMWL, Merck Millipore, Darmstadt, Germany) at 4500 × *g* for 30 min and incubated with ExoQuick overnight. Thereafter, the precipitate was resuspended in fresh DMEM for subsequent experiments. The supernatants collected from ExoQuick-incubated medium were named as exosome-free medium for further experiments.

### Electron microscopy

Electron microscopy was performed using a transmission electron microscope (JEM-12001 × ; JEOL, Tokyo, Japan) after standard staining procedures with phosphotungstic acid (Sigma-Aldrich, St. Louis, MO, USA).

### Cell viability assays

Cell viability was evaluated using the Cell Counting Kit-8 (CCK-8; Dojindo, Kumamoto, Japan). Cells were seeded onto ultra-low attachment 96-well plates (Corning, NY, USA) at 10,000 cells per well. After treatments, the cells were incubated in CCK-8 solution for 2 h, after which the absorbance was determined at 450 nm using a microplate reader (ELx808, BioTek, Winooski, VT, USA). Relative cell viability was expressed as a percentage of specific controls.

### Cell adhesion assays

To test the adhesive ability of HCC cells, 20000 cells were harvested and suspended in FBS-free medium, seeded on 96-well plates, and incubated for 2 h at 37 °C. The plates were then washed twice with phosphate-buffered saline (PBS), fixed with cold methanol for 30 min, and stained with 0.5% crystal violet dissolved in methanol for 1 h. The plates were further washed with PBS five times, and then photographed under a microscope (Leica, Wetzlar, Germany). The number of adhesive cells in five random fields was counted and normalized to that of the specific control.

To determine adhesive ability of HCC cells on the extracellular matrix and vascular endothelial cells, the 96-well plates were pre-coated with fibronectin (Roche; Basel, Switzerland) or HUVECs, respectively. For fibronectin coating, the plate was incubated with 100 μl of fibronectin solution (50 μg/ml) for 45 min at 25 °C and used immediately. For HUVECs coating, 20,000 HUVECs were seeded, and the plates were used when HUVECs reached 100% confluence.

### ROS determination

ROS was detected using a ROS Assay Kit (Beyotime Biotechnology, Shanghai, China) according to the manufacturer’s instructions. In brief, 1 × 10^6^ cells were suspended in FBS-free medium containing 0.1% Dichloro-dihydro-fluorescein diacetate (10 mM) and incubated for 20 min at 37 °C. After centrifugation, the precipitate was washed twice with PBS and resuspended in FBS-free DMEM. Fluorescence was measured using a microplate reader (SpectraMax Paradigm; Molecular Devices, Sunnyvale, CA, USA) with a 488 nm-excitation filter and a 525 nm-emission filter.

### Mouse model

Male nude mice aged 4–6 weeks were obtained from the Shanghai Experimental Animal Center and bred in a specific pathogen-free facility. The animal experimental protocol was approved by the Animal Care and Use Committee of the Second Affiliated Hospital, Zhejiang University School of Medicine (SAHZU; Hangzhou, China). The mice were allocated to experimental groups randomly. For xenograft inoculation, cancer cell suspensions (2 × 10^6^ cells/mouse) were injected subcutaneously. To generate CTCs, 1 × 10^6^ cells were injected through the caudal vein 2 weeks after subcutaneous inoculation of cancer cells. The mice were killed 4 weeks after intravenous injection of HCC cells. The gross morphology of the lungs and livers was examined for metastatic nodules. The mice groups were blinded to investigators during sample analyses.

### Evaluation of vascular permeability

Male nude mice, with or without subcutaneous xenograft inoculation (2 × 10^6^ cells/mouse), were injected with Evans Blue (EB, 20 mg/ml) to test vascular permeability. After 3 h of EB infusion, the lungs and xenografts were excised, and rinsed with PBS. The tissues were homogenized in PBS and incubated in formamide at 60 °C for 16 h. The absorbance of the supernatants at 620 nm and 740 nm were record. A_620_ was corrected for the presence of heme pigments according to the following formula: A_620corrected_ = A_620_−(1.426 × A_740_ + 0.030). The values were then compared with a standard curve of EB in formamide to calculate the tissue EB content.

### In vivo detection of GFP from PTDEs

Conditioned medium of GFP^+^ Huh-7 cells were collected to isolate exosomes containing GFP. Mice were intravenously injected with naive Huh-7 cells and GFP^+^ Huh-7-derived PTDEs successively. Mice were killed 2 h later, their lung tissue were grinded and lysed to get proteins for GFP detection.

### RNA purification, reverse transcription, and qRT-PCR

Total RNAs were isolated from cells using the Trizol reagent (Takara, Shiga, Japan). cDNA was synthesized by reverse transcription and subjected to quantitative real-time polymerase chain reaction (qPCR) with gene-specific primers in the presence of SYBR Green (Takara). The primers used are shown in Supplementary Table 1. QRT-PCR) was performed on an ABI 7900 Prism HT (Applied Biosystems, Foster City, CA, USA). Fold change in gene expression change was calculated using the comparative cycle threshold (CT) method. Each measurement was tested in triplicate and three independent experiments were performed.

### Stable transfection and Crispr/Cas9 genome editing

For stable transfection, Huh-7 cells were transfected using lentiviruses containing a sequence for overexpression of the FLAG-tagged SMAD3 protein. Cells were then cultured for 2 weeks in medium containing puromycin (1 μg/ml) to select stable transfectants. The colonies were isolated and named as FLAG-SMAD3 Huh-7. For Crispr/Cas9 genome editing, LentiCRISPR(pXPR_001) plasmids (a gift from Professor Yang Xiaohang at Zhejiang University School of Medicine) were digested with *Bsm*BI, and a pair of annealed oligonucleotides (Oligo 1: 5′-CACCGCCACCACGAGCTACGGGCCA-3′; Oligo 2: 5′-AAACTGGCCCGTAGCTCGTGGTGGC-3′) was cloned into the single guide RNA (sgRNA) scaffold, as described previously [32]. LentiCRISPR with the cloned sgRNA was transfected into Huh-7 cells. After 2 days of puromycin (1 μg/ml) selection, viable cells were collected and seeded onto a 96-well plate at a density of one cell per well. The *SMAD3*^−/−^ Huh-7 clones were confirmed by immunoblotting and DNA sequencing.

### Immunoblotting, immunofluorescence, and immunohistochemistry

These procedures were performed as previously described [33]. For immunoblotting, 20 µg of total proteins from cell lysates were loaded for gel electrophoresis. Proteins were transferred to a polyvinylidene difluoride membrane, which was then incubated overnight with primary antibodies after antigen blocking with non-fat milk. The membranes were then incubated with appropriate secondary antibodies and the signal was detected using an ECL Western Blotting Substrate Kit (Thermo Fisher Scientific). Primary antibodies used included those raised against SMAD3, cluster determinant 36 (CD63), glucose-regulated protein, 78 kDa (GRP78), GFP, and glyceraldehyde-3-phosphate dehydrogenase were from Cell Signaling Technology (Beverly, MA, USA). p-SMAD3 and tumor susceptibility gene 101 (TSG101) antibodies were from Epitomics (Burlingame, CA, USA).

For immunofluorescence, cells were treated as indicated and then fixed with cold 4% polyoxymethylene for 20 min. Cells were then incubated with Triton X-100 (Sigma-Aldrich) to increase their permeability. Following blocking with 1% bovine serum albumin, anti-FLAG antibodies (Huabio, Hangzhou, China) were incubated with the cells at room temperature for 1 h. Cyanine 3-conjugated secondary antibodies were then used, and the samples were observed using a fluorescence microscope (Leica).

For immunohistochemistry, formalin-fixed paraffin-embedded tissue samples were cut into 5-µm thick slices. Heat-induced antigen retrieval was performed using microwave (in sodium citrate, PH 6.0, 98 °C, 10 min). The slides were then incubated with anti-SMAD3 antibodies (Ruiying Biological, Suzhou, China) and corresponding horseradish peroxidase-conjugated secondary antibodies. The slides were stained using a Histostain-Plus Kit (ZSGB-BIO, Beijing, China) and then further counterstained with hematoxylin.

### Enzyme-linked immunosorbent assay (ELISA)

Exosomes were extracted from the serum of patients. After three cycles of freezing and thawing, proteins within the exosomes were released into the supernatant. The SMAD3 protein concentration was determined using a specific ELISA kit (Mlbio, Shanghai, China) following the manufacturer’s protocol.

### Acquisition of human specimens

Patients with HCC and patients with benign hepatoma were recruited at the Department of Hepatobiliary and Pancreatic Surgery, SAHZU, between January 2016 and December 2016. Healthy donors were recruited at the Physical Examination Center of SAHZU in May 2016. Serum samples were isolated and kept in −80 °C until used. HCC samples were acquired intra-operatively and were frozen in liquid nitrogen for 30 min before transferred to −80 °C. The protocol of this study was approved by the ethical committee of SAHZU. All patients provided written informed consent.

### Statistical analysis

The data are presented as the mean ± standard deviation or standard error of the mean. Student’s *t* test or one-way analysis of variance were used for statistical analysis, as appropriate. Survival data were estimated using the Kaplan–Meier method and compared using the long-rank test. Data analysis was performed using Prism software (version 6; GraphPad, San Diego, CA, USA) and SPSS software (version 24; SPSS, Chicago, IL, USA). A *p* value < 0.05 was considered statistically significant.

## Electronic supplementary material

Table S1
