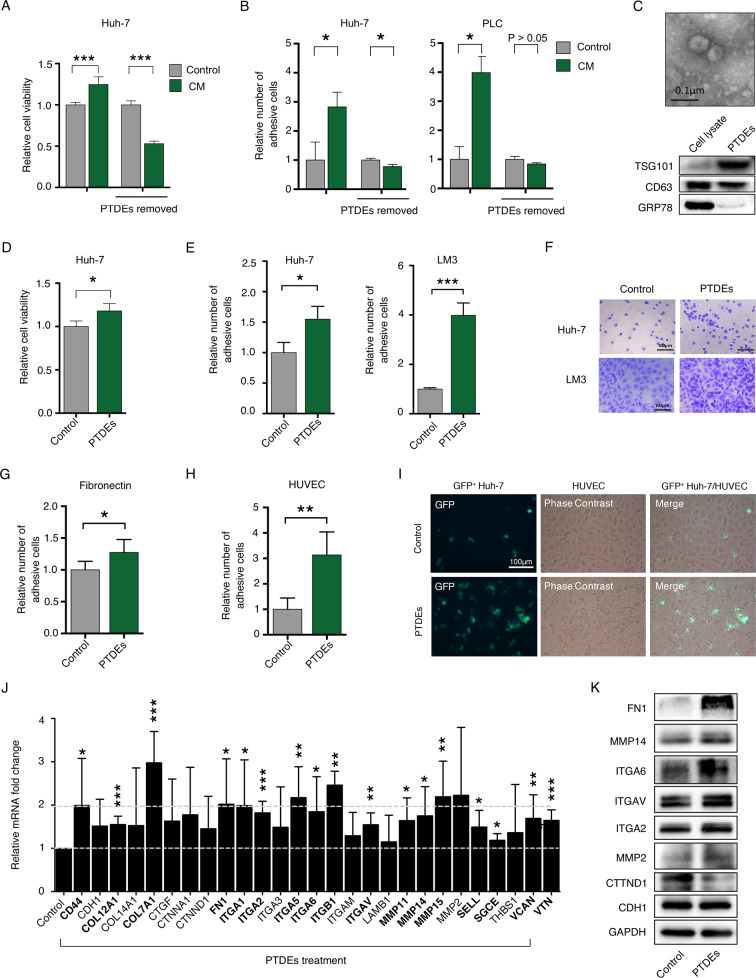# Correction: Primary tumor-derived exosomes facilitate metastasis by regulating adhesion of circulating tumor cells via SMAD3 in liver cancer

**DOI:** 10.1038/s41388-019-0830-6

**Published:** 2019-05-08

**Authors:** Qihan Fu, Qi Zhang, Yu Lou, Jiaqi Yang, Gang Nie, Qi Chen, Yiwen Chen, Jingying Zhang, Jianxin Wang, Tao Wei, Hao Qin, Xiaowei Dang, Xueli Bai, Tingbo Liang

**Affiliations:** 1 Department of Hepatobiliary and Pancreatic Surgery, the Second Affiliated Hospital, Zhejiang University School of Medicine, Hangzhou, China; 2 Zhejiang Provincial Key Laboratory of Pancreatic Disease, Hangzhou, China; 3 Department of Medical Oncology, the Second Affiliated Hospital, Zhejiang University School of Medicine, Hangzhou, China; 4 Department of Hepatopancreatobiliary Surgery, the First Affiliated Hospital of Zhengzhou University, Zhengzhou, China

**Keywords:** Metastasis, Oncogenes

**Correction to: Oncogene** 37:6105–6118

10.1038/s41388-018-0391-0 published online 10 July 2018

Following publication of this article the Authors noted mislabelling in Fig. 2k. The label ITGA2 had been duplicated and CDH1 had been omitted. The correct version of the figure is included below.

**Figure 1:**