# Supplementary material for: Primary tumor-derived exosomes facilitate metastasis by regulating adhesion of circulating tumor cells via SMAD3 in liver cancer
Source: Oncogene. 2018 Jul 10;37(47):6105–18. doi: 10.1038/s41388-018-0391-0 (PMC6250679; doi:10.1038/s41388-018-0391-0)
Supplement: Supplementary file 1 — Table S1 [file 41388_2018_391_MOESM1_ESM.pdf]

Table S1 Primers

Gene Name	Forward Primer(5'-3')	Reverse Primer(5'-3')
CD44	TGGCAGCCCCGATTATTTACA	GACCTAAGACGGAGGGAGGG
CDH1	CAAAGCACCTGTGAGCTTGC	TGTAGCTCTCGGCGTCAAAG
COL12A1	GAACGAGGTTTGCCAGGAGA	ATGTGTTAGCCGGAACCTGG
COL14A1	CTCCGAGGGAAGAGAGCAAG	AGCAACCAGTACCGCATCTT
COL7A1	TTACGCCGCTGACATTGTGTT	ACCAGCCCTTCGAGAAAGC
CTGF	ACCGACTGGAAGACACGTTTG	CCAGGTCAGCTTCGCAAGG
CTNNA1	TCCGATCCTCTATACTGCATCC	TGCTGTGAGGCATCGTCTG
CTNND1	ATGAAGATGGTTATCCAGGTGGC	CTGGGCCTATACCGCTCCT
FN1	GAGAATAAGCTGTACCATCGCAA	CGACCACATAGGAAGTCCCAG
ITGA1	GTGCTTATTGGTTCTCCGTTAGT	CACAAGCCAGAAATCCTCCAT
ITGA2	GGGAATCAGTATTACACAACGGG	CCACAACATCTATGAGGGAAGGG
ITGA3	TGTGGCTTGGAGTGACTGTG	TCATTGCCTCGCACGTAGC
ITGA5	GGCTTCAACTTAGACGCGGAG	TGGCTGGTATTAGCCTTGGGT
ITGA6	GAGGAATATTCCAAACTGAACTAC	GGAATGCTGTCATCGTACCTAGAG
ITGB1	AGGATTACTTCGGACTTCAGA	CTTTGGCATTCACATTCA
ITGAM	ACTTGCAGTGAGAACACGTATG	TCATCCGCCGAAAGTCATGTG
ITGAV	GCTGTCGGAGATTTCAATGGT	TCTGCTCGCCAGTAAAATTGT
LAMB1	CACAAGCCCGAACCCTACTG	GACCACATTTTCAATGAGATGGC
MMP11	AAGGTATGGAGCGATGTGACG	GTCCAGGTCTCATCATAGTCGAA
MMP14	CGAGGTGCCCTATGCCTAC	CTCGGCAGAGTCAAAGTGG
MMP15	GTGCTCGACGAAGAGACCAAG	TTTCACTCGTACCCCGAACTG
MMP2	CCCACTGCGGTTTTCTCGAAT	CAAAGGGGTATCCATCGCCAT
SELL	TGCCTTTCAGTCGTTCTTACTAC	GGCGTCATCGTTCCATTTGC
SGCE	AACATCACATCGGCCCTAGAC	AACCATGACATAAACGCCCTC
THBS1	GCCATCCGCACTAACTACATT	TCCGTTGTGATAGCATAGGGG
VCAN	GCAAGTGATGCGGGTCTTTAC	TTGCCGCCCTGTAGTGAAAC
VTN	GCCTTCACCGACCTCAAGAAC	CCCCTGACAGTTGATGCGG
Smad3	AGGCGTGCGGCTCTACTACATC	CAGCGAACTCCTGGTTGTTGAA
Flag-Smad3	TGTCGTCCATCCTGCCTTTC	GTCGTCATCATCCTTATAGTCCTT
